# Editorial: Utilization of crop wild relatives for trait discovery for climate-smart crops

**DOI:** 10.3389/fpls.2023.1231825

**Published:** 2023-06-27

**Authors:** Dorin Gupta, K. C. Bansal

**Affiliations:** ^1^ School of Agriculture, Food and Ecosystem Sciences, Faculty of Science, The University of Melbourne, Dookie College, Australia; ^2^ National Academy of Agricultural Sciences, New Delhi, India

**Keywords:** crop wild relatives (CWR), climate change, biotic and abiotic stress, climate smart crops, diversity

This Research Topic synthesizes different contributions highlighting the role of crop wild relatives (CWRs) that possess valuable traits for improving and enhancing the adaptation of current commercial crop cultivars to the fast-changing climate. However, their optimal utilization is challenged at the very basic level because of a lack of essential information on CWR accessions in genebanks and subsequent lack of their utilization in respective crop breeding programs. To fully employ CWRs, the public and private sectors need to be better coordinated with enabling policies, proper funding support, and adequate capability building. In addition, there is a need for the execution of systematic data-driven and informed germplasm collection and management. Furthermore, breeding, genomics, and gene-editing innovations present fascinating opportunities for the timely employment of CWRs in breeding programs. Ultimately, climate-smart crops will ensure food and nutrition security and the proper use of CWRs will especially benefit farmers in tropical and subtropical countries.

Our concern is that climate change will very likely affect crop productivity, especially that of staple crops (IPCC, 2022). Wheat is a crop that is extremely sensitive to temperatures outside of its ideal range. Researchers have started using CWRs to tackle this problem and enhance wheat tolerance to heat stress.

The first article included in this Research Topic, by Yang et al., has provided valuable insight into the diversity and evolution of transcription factor (TF) genes in wheat, which can be used for its genetic improvements. Specific TF genes regulate gene expression, which ultimately determines the related phenotype. The authors successfully identified a total of 6,023 TF genes grouped into 59 gene families and performed a comprehensive genome search, followed by a classification process. A major finding from their study was the significance of these genes for the agronomical traits of wheat crop. The related characteristics and dN/dS values of these TF genes are linked to their selective effects. Interestingly, using information based on selective signals and known quantitative trait loci (QTLs), 21 TF genes were eventually linked with yield-related traits. An important insight was that selective pressure due to domestication on the A and D sub-genomes could potentially be responsible for a genetic bottleneck in TF genes. Wheat breeding programs aim to increase gene diversity while developing climate-smart cultivars. These programs now have these TF genes and their accompanying haplotypes linked to critical characteristics.

The valuable insights into the potential of utilizing CWRs of wheat for trait discovery in developing climate-smart cultivars are further strengthened by Balla et al. They focused on identifying heat stress tolerant wild emmer wheat germplasm and associated QTLs. Multiple derivative lines of nine wild parents from different heat stress environments and genome-wide association analysis using SNP markers identified strong marker-trait associations (MTAs). Interestingly, various MTAs on 1A, 3A, 3B, and 5B chromosomes revealed an association with useful traits, such as chlorophyll content at plant maturity (1A and 5B chromosomes), traits related to grain yield (3A and 3B), biomass (3A and 3B), days to maturity (3A), thousand kernel weight (3A), and heat tolerance efficiency (2A, 2B, 3A, and 5A chromosomes) under different heat stress levels. Importantly, now we know that some of the heat stress tolerance-linked favorable alleles are missing or are rare in the elite durum wheat germplasm. Therefore, the work of these authors highlighted the potential of using the identified lines, MTAs, and QTLs from wild emmer wheat in breeding programs to improve wheat adaptation to heat stress. Furthermore, the identified germplasm lines hold promise for enhancing tolerance to other stressors leading to the development of multiple stress-resistant, climate-smart crops.

Sustaining crop production also includes climate-smart cropping options for animal feed. In this context, important work published in this Research Topic included a study by Christiansen et al. on the perennial halophytic shrubs *Atriplex nummularia* and *Rhagodia preissii*. These shrubs can potentially be grown on saline land in Australia as feed for sheep and cattle during autumn. These researchers filled the critical knowledge gap on optimal germination conditions, which up until now had seen limited adoption of direct seeding techniques. The research showed that these shrubs can be optimally germinated at 10°C, whereas *Atriplex nummularia* seeds seem more tolerant to higher levels of stressors (temperature and salinity). The study recommended the use of these underutilized shrubs through direct seeding and by sowing them during the cold winter months (subtropical regions) or in autumn and spring (temperate regions) for their successful establishment in the saline lands of Australia.

The next research on this Research Topic is presented by Charles et al., who provide insights into sustainable and efficient climate-smart crop production systems using semi-transparent organic solar cells (ST-OSCs) in greenhouses. Three different ST-OSC filters with varying light spectra affected both the power generation and crop growth of lettuce and tomato plants. Integration of ST-OSCs into greenhouses offers opportunities for energy-neutral and climate-protected crop production. Such benefits go beyond energy considerations; they could potentially enhance the nutritional value of crops and influence their growth and development patterns, such as flowering initiation in tomatoes.

While the first and second studies included in this Research Topic focused on the wheat crop, one of the main and well-researched staple crops, the final study, by Rajpal et al., focuses on the lentil crop, one of the less-known and researched crops. Modern lentil varieties, similar to other staple crops, also struggle with a narrow genetic base due to the loss of their genes during recurrent selections and genetic bottlenecks. However, recent advances in the collection and characterization of lentil CWRs have provided useful avenues for developing improved lentil cultivars that possess tolerance to various stresses, including being climate smart. To have an efficient and effective breeding program for the identification and introgression of desirable traits from distant and CWRs in lentils requires the selection of such traits using related QTL identification and marker-assisted selection (MAS). Recent advancements in genetic diversity studies, genome mapping, and high-throughput sequencing have facilitated the identification of stress-responsive adaptive genes, QTLs, and other favorable traits in CWRs of lentils. Integration of genomic technologies with conventional plant breeding technologies has paved the way for developing dense genomic linkage maps, global genotyping, and large transcriptomic datasets, enabling MAS and breeding in lentils. The lentil genome assembly and exploration of its wild species have provided novel insights into the genomic architecture and evolution of this less researched and understood legume crop.

In the context of climate change and the compelling race to develop climate-smart cultivars, it is critical to note the advancements made in characterizing wild genetic resources, developing high-density genetic maps, carrying out precise QTL mapping, performing genome-wide association analyses, and applying genomics technologies for lentil improvement.

These developments support climate resilience in different crops to fulfill future food demand and secure sustainable crop yields. Researchers and breeders can combine genetic traits and rare genes and alleles from wild crop relatives to create climate-smart crops that could guarantee food security.

We hope readers will find this Research Topic and published research papers critical in filling the existing knowledge gaps in utilizing CWRs in crop improvement through data-informed germplasm collection and their utilization for developing climate-smart crops.

## Author contributions

DG and KB contributed to the editorial write up. DG wrote the first draft and KB edited the draft. Both the authors read, and approved the submitted version.
